# Mycorrhizal fungi volatiles: determining the fate of plants against stress?

**DOI:** 10.3389/fpls.2026.1756587

**Published:** 2026-02-24

**Authors:** Esperanza Miñambres, María Chaparro-Arias, Jorge Señorans, Sara Valera-León, Ainhoa Soria-Solabarrieta, Mónica Calvo-Polanco

**Affiliations:** Institute for Agribiotechnology Research (CIALE), Research Unit of Excellence “Agricultural Production and Environment” (AGRIENVIRONMENT), University of Salamanca, Villamayor, Salamanca, Spain

**Keywords:** abiotic stress, biotic stress, mycorrhizal fungi, plant development, volatile organic and inorganic compounds

## Abstract

Mycorrhizal fungi represent one of the oldest and most successful symbioses in plant evolution. Communication among mycorrhizal fungi and plants occurs prior to direct contact among them through different and variable biochemical signals, including microRNAs, hormones, small peptides and volatile organic and inorganic compounds. Volatile organic compounds (VOCs) emerge as key chemical signals that enable the transmission of chemical messages modulating plant and microorganism responses in both below- and above-ground ecosystems. The diversity and concentration of mycorrhizal VOCs will vary depending on the environment and the emitting organism and are usually related to changes in the conformation of root architecture and lateral root formation mediated by auxin and strigolactones. Moreover, the study of the effects of mycorrhizal VOCs in the tolerance to abiotic and biotic stress are still scarce although there are some promising results pointing out to the effect of these VOCs in plant development under osmotic stress conditions, and their properties as antifungal and antibacterial molecules. However, the information regarding the molecular mechanisms involved in mycorrhizal VOCs signaling and their effect on plants remains still elusive. The understanding of VOC-mediated plant-mycorrhizal interactions, together with the technical improvements for their detection and mode of application in the field, will open new avenues for biotechnological crop improvement and management that not only will reduce the dependence on agrochemicals but also fosters soil health and plant resilience.

## 1 Introduction

The rhizosphere constitutes a soil microhabitat that promotes the development of specific microbial and plant communities associations, influencing nutrient and water mobilization, soil structure and its ecological functions (Deveau, 2016; Solomon et al., 2024; Luo et al., 2025). Mycorrhizal fungi represent one of the oldest and most successful symbioses in plant evolution, with evidence tracing their origins back more than 400 million years, being decisive in the terrestrial colonization by vascular plants (van der Heijden et al., 2015). The main groups of mycorrhizal fungi, arbuscular mycorrhizal (AMF) and ectomycorrhizal (ECM) fungi, establish symbiotic associations with approximately 96% of vascular plants (agricultural and forestry), playing a fundamental role in plant survival and growth, increasing the tolerance of plants to adverse environmental conditions and modulating defense mechanisms against pathogens (Pandey et al., 2019; Drijber and McPherson, 2021). In exchange, mycorrhizal fungi obtain from the plant carbon compounds derived from photosynthesis—primarily sugars and lipids—which constitute their main energy source (Kiers et al., 2011; Becquer et al., 2019; Drijber and McPherson, 2021).

**Figure 1:**
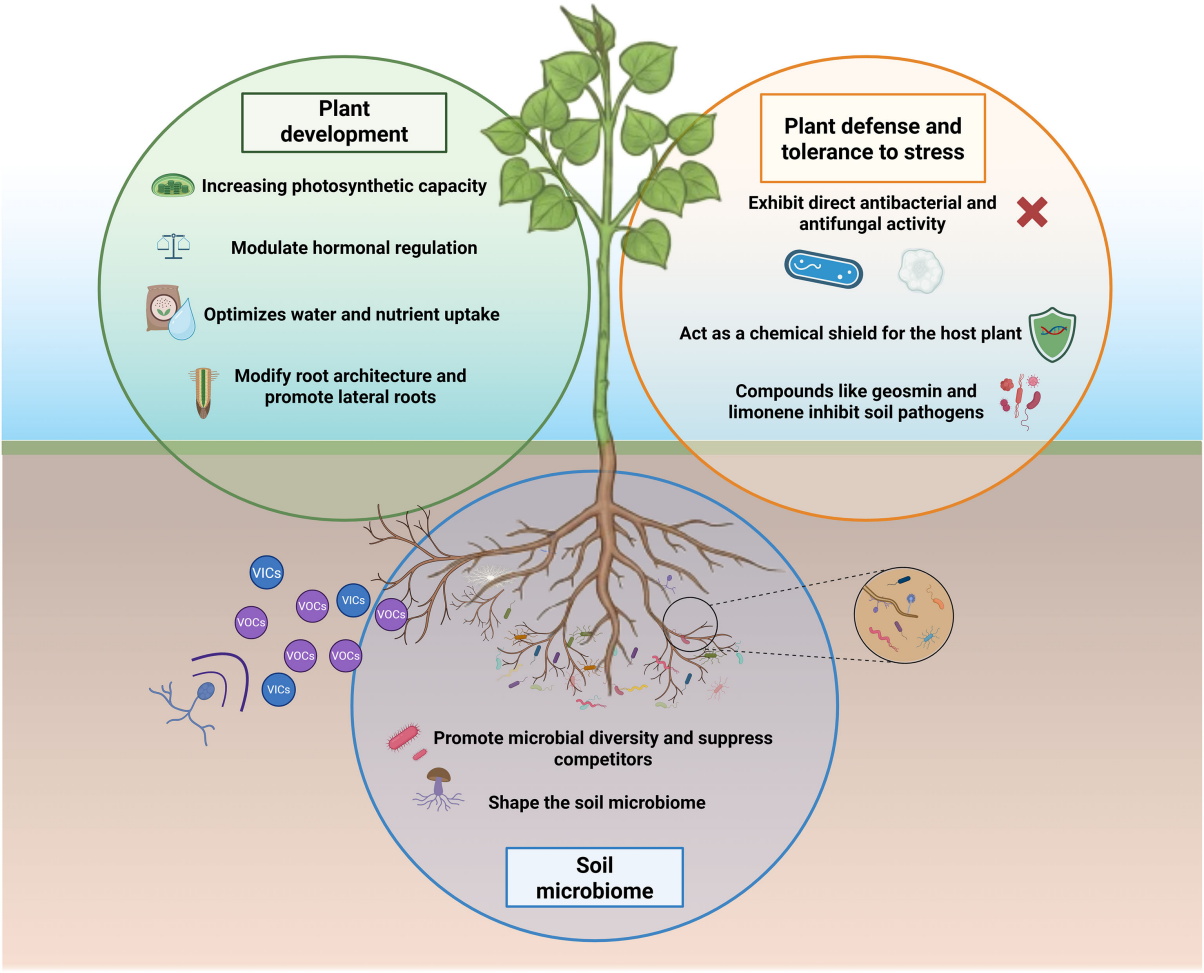
Main effects of mycorrhizal VOCs in plant development, plant defense and tolerance to stresses and soil microbiome configuration.

Communication among mycorrhizal fungi and plants occurs even prior to direct contact among them through different and variable biochemical signals, including microRNAs (Ledford et al., 2024), hormones (Pons et al., 2020), small peptides (Valmas et al., 2023) and volatile organic compounds (VOCs) (Plett et al., 2024) and volatile inorganic compounds (VICs) (Venneman et al., 2020; Sun et al., 2024). Mycorrhizal fungi release a wide spectrum of VOCs into the rhizosphere that are expected to interact with the composition and dynamics of soil microbial communities (Abis et al., 2020), affecting pathogen control (Gabriel et al., 2018) and plant development and tolerance (Ferreira et al., 2023; Inamdar et al., 2020; Sharifi et al., 2022; Türksoy et al., 2025) (**Figure 1**).

Although the effects of mycorrhizal fungi VOCs are expected to be relevant not only for plant development (Ditengou et al., 2015) but also for their role in plants against abiotic (Laller et al., 2023) and biotic stresses (Moisan et al., 2020; Razo-Belmán et al., 2023), the studies in this field are still limited. The understanding and harnessing of VOC-mediated plant-mycorrhizal interactions will open new avenues to improve crop performance and manage environmental stress under changing climatic conditions, more considering the diversity of the mycorrhizal fungi and their key roles both in agricultural forestry crops, together with their high relevance in ecosystem functioning. Within this review, we will focus on how mycorrhizal VOCs mediate plant development and modulate the responses to biotic and abiotic stresses, and we will cover the different biotechnological technologies available for the application of VOCs in agricultural systems.

## 2 Volatile compounds

### 2.1 Volatile organic and inorganic compounds

**Figure 2:**
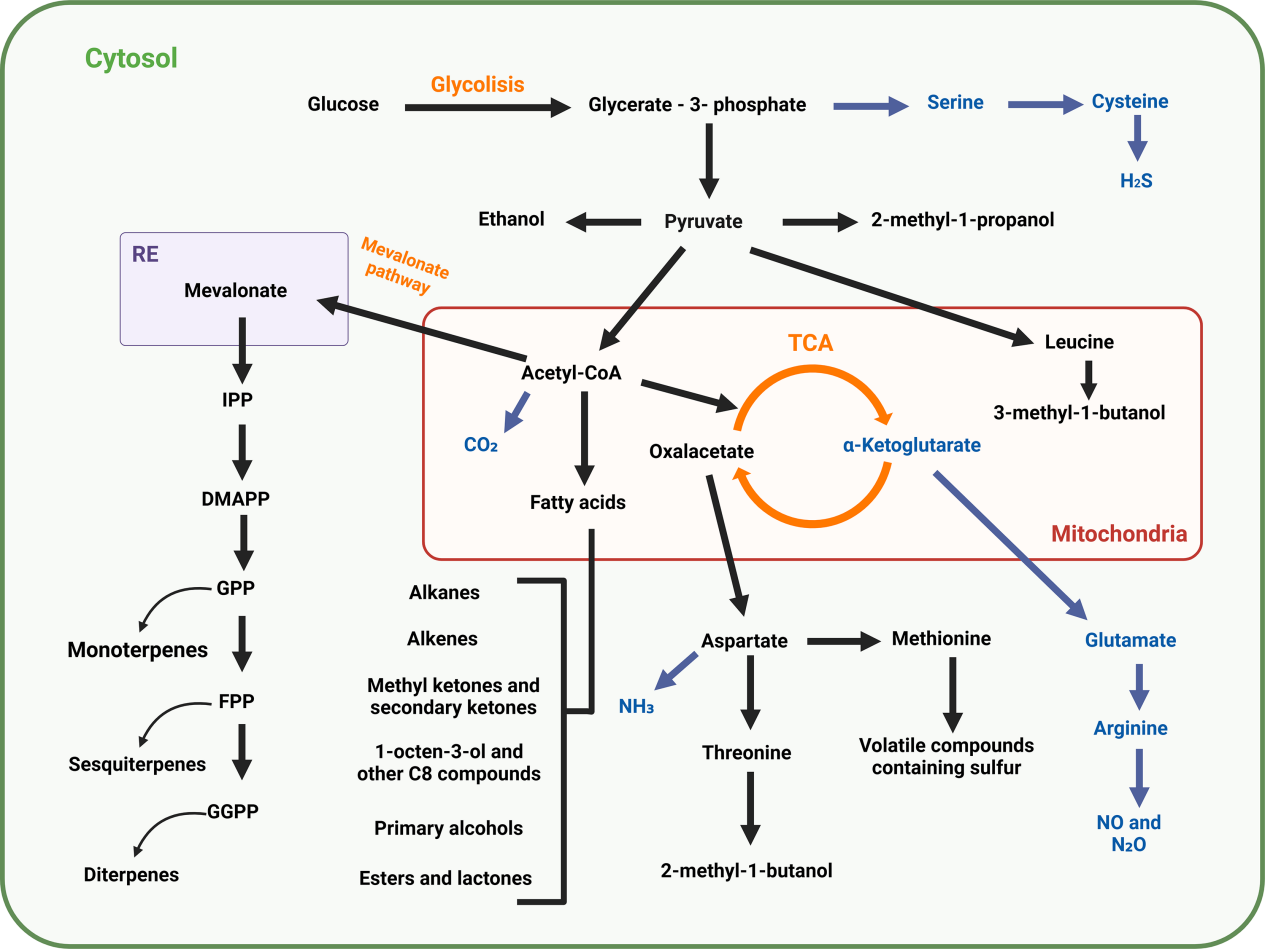
Main biosynthesis routes of volatile organic (black arrows, VOCs) and inorganic (blue arrows, VICs) compounds in mycorrhizal fungi.

The volatilome of animals, bacteria, fungi and plants is defined by a wide repertoire of volatile organic (VOCs) and inorganic (VICs) compounds (Ledford and Meredith, 2024) that are closely linked to ecosystem communication networks and play crucial roles in ecological interactions (Effmert et al., 2012; Batty et al., 2024). VOCs can include fatty acid derivatives (hydrocarbons, ketones, alcohols), acids, sulfur- and nitrogen-containing compounds and terpenes (El Jaddaoui et al., 2023; Yin et al., 2025). VOCs are carbon-based, low weight molecules that can easily evaporate at standard temperature and pressure (0.1 kPa 20 °C) (Inamdar et al., 2020) that can be generated from both primary and secondary metabolism, resulting in a wide variety of compounds (Morath et al., 2012) (**Figure 2**). On the other hand, VICs include hydrogen sulfide (H_2_S), a gasotransmitter that regulates developmental cues and enhances abiotic stress tolerance in plants (Corpas and Palma, 2020; Yang et al., 2022); nitrogen oxides (NO and N_2_O) involved in plant growth, defense signaling (Hancock, 2020) and abiotic stress tolerance (Mata-Pérez et al., 2023); ammonia (NH_3_), essential for plant growth as a source of nitrogen (Paloyan and Dyukova, 2024); and carbon dioxide (CO_2_), essential for plant and fungal growth (Audrain et al., 2015) (**Figure 2**). Despite the relevance of microorganisms VICs in plant development, studies of volatiles have mainly focused on microorganisms and plant VOCs as the main communication pathways between plants and microorganisms.

Mycorrhizal fungi emit a diverse array of VOCs (**Table 1**) (Guo et al., 2021; Duc et al., 2022; Ferreira et al., 2023) that can both positively and negatively affect plant development (Ditengou et al., 2015). The proportion and concentration of each volatile emitted by the different mycorrhizal fungi will depend on factors such as the substrate where they grow, the humidity level, temperature and the developmental stage of the mycorrhizal fungi, among others (El Jaddaoui et al., 2023). Based on this fact, the description of the main factors affecting key VOCs emitted by fungi can be a key tool to understand the main effects on plants. However, despite the limitations of the current measurement techniques to detect VOCs currently available, the lack of knowledge about the complete biosynthetic pathways from where they are generated (**Figure 2**), and the diverse fungal growth conditions used in different experiments would need to be improved to obtain meaningful results.

**Table 1:** Mycorrhizal fungal volatile organic compounds.

Mycorrhizal fungi	Host species	Fungal VOCS	Mechanisms	Reference
*Amanita porphyria*	*Quercus* sp.	3,4-Dimethyl-2-hexanone; 4-Methoxyphenylacetic acid ethyl ester; 4-methyl ester; methyl cinnamate	Plant defense and growth	Guo et al. (2021)
*Boletus reticulatus*	*Cistus* spp.*; Halimium halimifolium;* *Tuberaria guttata*	1-octen-3-ol	Root architecture and lateral root	Ferreira et al. (2023)
*Cenococcum geophilum*	*Populus tremula x alba* *Arabidopsis thaliana*	3-carene	Plant growth	Ditengou et al. (2015)
*Cortinarius glaucopus*	*Quercus* sp. *Abies* sp. *Picea* sp. *Larix* sp. *Cedrus* sp. *Corylus avellana*	α-selinene; 3-heptanone,4-methyl-	Plant defense and growth	Guo et al. (2021)
*Glomus mosseae*	*P. vulgaris*	β-ocimene; β-caryophyllene; methyl salicylate	Plant defense	Schausberger et al. (2012)
*Laccaria bicolor*	*Pinus* sp.	Bicyclogermacrene	Plant defense and growth	Guo et al. (2021)
	*Populus tremula x alba* *Arabidopsis thaliana*	3-carene; (-)-thujopsene	Plant and root growth	Ditengou et al. (2015)
*Lactarius deliciosus*	*Cistus* spp.*; Halimium halimifolium;* *Tuberaria guttata*	1-octen-3-ol	Root architecture and lateral root	Ferreira et al. (2023)
*Meliniomyces bicolor*	*Erica* sp.	1,3-Dimethoxybenzene	Plant defense and growth	Guo et al. (2021)
*Piloderma croceum*	*Picea* sp. *Pinus* sp.	3-Octanol; 1-Octen-3-ol; 3-Octanone	Plant defense and growth	Guo et al. (2021)
*Pisolithus microcarpus*	*Eucalyptus grandis*	γ-cadinene	Host colonization and root growth	Plett et al. (2024)
*Rhizophagus irregularis*	*Medicago truncatula*	Limonene; β-pinene; Nerolidol	Plant growth and defense	Dreher et al. (2019)
*Terfezia arenaria*	*Cistus salviifolius*	1-octen-3-ol	Root architecture and lateral root	Ferreira et al. (2023)
*Terfezia leptoderma*	*Cistus* spp.*; Halimium halimifolium;* *Tuberaria guttata*	1-octen-3-ol	Root architecture and lateral root	Ferreira et al. (2023)
*Tomentellopsis zygodesmoides*	*Pinus* sp. *Picea* sp.	2-Ethylhexanol; α-Selinene	Plant defense and growth	Guo et al. (2021)
*Tricholoma vaccinum*	*Picea abies*	Geosmin	Soil microbiota	Abdulsalam et al. (2021)
*Tuber borchii*	*Arabidopsis thaliana*	1-hexanol; 3-octanol; 1-Octen-3-ol; trans-2-Octenal; 3-Octanone	Growth inhibition	Splivallo et al. (2007)
	*Tilia americana*	5β,6β-epoxy-7α-bromocholestan-3β-ol	Plant and root growth and defense	Menotta et al. (2004)
*Tuber indicum*	*Arabidopsis thaliana*	1-hexanol; 3-octanol; 1-Octen-3-ol; trans-2-Octenal; 3-Octanone	Growth inhibition	Splivallo et al. (2007)
*Tuber melanosporum*	*Arabidopsis thaliana*	1-hexanol; 3-octanol; 1-Octen-3-ol; trans-2-Octenal; 3-Octanone	Growth inhibition	Splivallo et al. (2007)

### 2.2 Biosynthetic pathways of volatile organic compounds

The main pathways involved in the synthesis of fungal VOCs include glycolysis (yielding pyruvate and acetyl-CoA) and the Krebs cycle or tricarboxylic acid (TCA) cycle (producing oxaloacetate) (**Figure 2**). Some alcohol-type VOCs compounds are derived from pyruvate, while acetyl-CoA gives rise to mevalonate, which is the precursor for terpenoid volatile compounds and fatty acids, the latter being precursors for numerous VOC types. As for oxaloacetate, it serves as the precursor for aspartate and methionine, which in turn give rise to other types of VOCs, such as alcohols and sulfur-containing compounds (Schnürer et al., 1999; Pichersky and Lewinsohn, 2011; Dudareva et al., 2013; Kaddes et al., 2019; Chen and Liao, 2025).

The study of VOCs lags behind other metabolites due to limitations in detection and isolation methods (Heffernan et al., 2023). Gas chromatography coupled with mass spectrometry (GC-MS) is widely used due to its high sensitivity for separating and detecting compounds, although its ability to identify previously undescribed molecules is limited (Elke et al., 1999; Butkovich et al., 2025). Other types of spectrometry are also used to detect new compounds, such as selected ion flow tubes (Scotter et al., 2005) or proton-transfer-reaction mass spectrometry (Ezra et al., 2004). Meanwhile, traditional methods such as simultaneous distillation, steam distillation and solvent extraction are insufficient for VOC profiling (Morath et al., 2012). Activated carbon filters are useful for detecting hydrocarbons, ethers, alcohols, and ketones but are less efficient for compounds like amines, phenols, aldehydes, and unsaturated hydrocarbons (Matysik et al., 2009; Morath et al., 2012). A promising recent development for detecting VOC profiles is the electronic nose, which combines a multisensor array with an information processing unit, pattern recognition software and a reference library (Li et al., 2024). Finally, the mVOC 4.0 database (https://bioinformatics.charite.de/mvoc/; Kemmler et al., 2025) contains data on thousands of described microbial volatiles, which can be a precious tool for the understanding of VOCs emitted by different mycorrhizal fungi species.

### 2.3 Plant perception of volatile organic compounds

The entry of VOCs in plants occurs mainly by passive diffusion through the epidermis and cell walls, facilitated by the lipophilic nature of many VOCs, as well as through membrane receptor-mediated processes (Razo-Belmán et al., 2023; Bergman et al., 2025). These processes have been little studied in mycorrhizal fungi and are more common in plant-to-plant communication or in interactions in the presence of bacterial VOCs. Nevertheless, in general, VOCs will induce the production of reactive oxygen species (ROS), calcium influx and nitric oxide (NO) signaling in plants. These responses will be integrated into signaling pathways such as MAPK cascades, essential for regulating physiological processes like defense, photosynthesis, metabolism, nutrient balance and hormonal interactions (Razo-Belmán et al., 2023). Inside the cell and in response to stress, VOCs may interact with specific proteins such as TOPLESS (TPLs) transcription factors, which bind the sesquiterpene caryophyllene (Nagashima et al., 2019). If these genes are main true fungal VOCs receptors it is still unknown, although it is hypothesized that the presence of specific membrane receptors in plant cells that recognize beneficial fungal signals and activate molecular pathways compatible with symbiosis would be main target for future studies (Bergman et al., 2025).

## 3 Effects of mycorrhizal VOCs in root architecture and lateral root formation

Mycorrhizal fungi have been well documented for their influence on root architecture and lateral root formation (Fusconi, 2014; Chiu et al., 2022). The formation of lateral roots induced by mycorrhizal fungi often occurs through the strigolactone signaling pathways (López-Ráez et al., 2026), which at the same time interacts with auxin, the main hormone responsible for controlling the balance between cell division and differentiation in the root meristem and regulate lateral root formation (Chandler and Werr, 2015; Chen et al., 2017).

The promotion of lateral roots is among the main effects observed in plants subjected to mycorrhizal VOCs (**Figure 1**). Felten et al. (2010) and Splivallo et al. (2009) were among the first to indicate that VOCs emitted by ectomycorrhizal fungi *L. bicolor* can specifically activate the root developmental program in host plants through modulation of plant auxin and ethylene pathways, while Sun et al. (2015) demonstrated that the *Gigaspora margarita* VOCs increased the formation of lateral roots via the activation of genes related to strigolactone biosynthesis (**Table 1**). The molecular regulation underlying the promotion of lateral roots governing direct mycorrhizal fungi colonization vs. mycorrhizal VOCs - induced plants takes place in an intricate hormonal crosstalk where auxin and strigolactones are prevalent (Arora et al., 2024). Hence, inoculated plants have been described to have events of massive auxin accumulation (Felten et al., 2009), or to induce the suppression of the peptide CEP2 to release the brakes on auxin signaling (Hsieh et al., 2022), while VOCs exposed plants regulated auxins involving Pin-type polar transporters through the presence of sesquiterpene (SQTs) (–)-thujopsene (Ditengou et al., 2015). As for strigolactones, the presence of mycorrhizal fungi induces a tight management of the negative regulators SMAX1, related to strigolactone biosynthesis (Choi et al., 2020), while mycorrhizal VOCs seem to bypass this complex suppressor pathway directly upregulating biosynthetic strigolactones genes like *LjCCD7* (Sun et al., 2015). The effects of mycorrhizal VOCs in root formation is, nevertheless, species dependent, as no effects were also reported in *P. tremula x alba* seedings under the VOCs of the ECM fungi *Cenococcum geophilum* (Ditengou et al., 2015), *Tuber borchii* VOCs and *Tilia americana* (Menotta et al., 2004) and the VOCs from *Tuber melanosporum*, *Tuber indicum* and *Tuber borchii* in Arabidopsis (Splivallo et al., 2007), showing the complex and diverse relationship between mycorrhizal VOCs and plants that will need further information in order to be completely elucidated.

In summary, how mycorrhizal VOCs can emerge as essential signaling molecules in the communication with plants will be only achieved with the study of the main molecules and mechanisms behind their effects on plants, as these molecules are expected to synergistically modulate plant responses and will contribute to the multifaceted chemical dialogue that orchestrates symbiotic interactions and adaptations of plants to their environment.

## 4 Role of mycorrhizal VOCs on abiotic stress tolerance

Plants have to face multiple environmental restrictions over their life cycle. Abiotic stresses such as drought and salinity are among the main concerns for ecosystems sustainability and crop production (European Environment Agency, 2025; FAO, 2024). These stresses affect plant development and productivity in agricultural and forestry systems globally, impacting molecular, biochemical, morphological and physiological processes depending on age, species and the severity of the stress (Seleiman et al., 2021; Pandit et al., 2024). Direct interactions among plants and mycorrhizal fungi have been extensively studied in different agricultural and forestry species for their crucial role in mitigating the detrimental effects of drought (Valenzuela-Aragon et al., 2025) and salt stress (Zwiazek et al., 2019) by improving water and nutrient uptake (Calvo-Polanco et al., 2019; Li et al., 2021; Fresno et al., 2023) and activating different hormonal and molecular mechanisms (Ye et al., 2023; Guarnizo et al., 2023; Cao et al., 2025).

**Figure 3:**
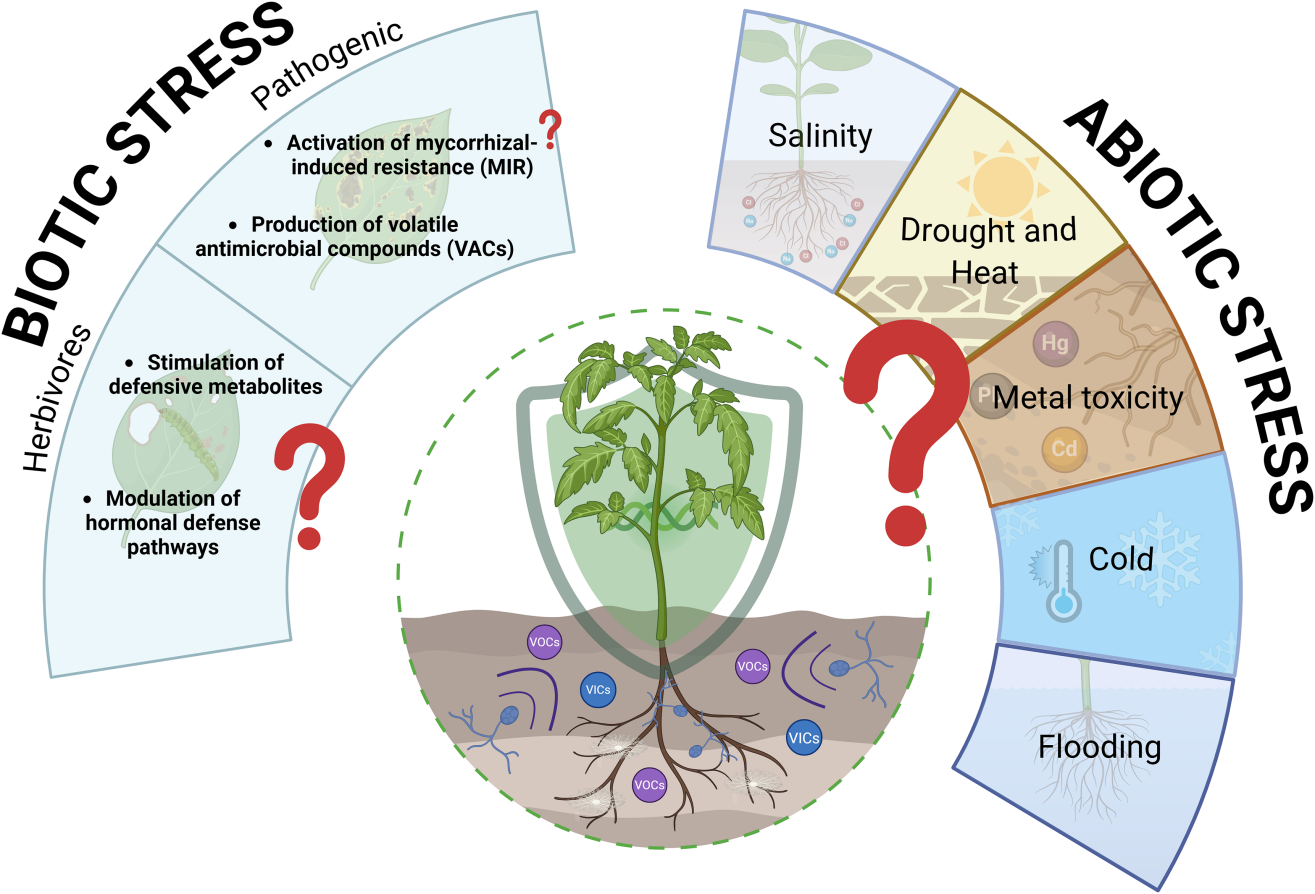
Abiotic and biotic stress tolerance of plants induced by mycorrhizal fungi VOCs. Abiotic stresses such as salinity, flooding, and heat and cold stresses are not studied yet related to mycorrhizal VOCs.

The main information on the effects of mycorrhizal VOCs on plant responses under abiotic stress (**Figure 3**) comes from a recent experimental study by Miñambres et al. (2025). This study showed that the exposure of Arabidopsis and *Populus tremuloides* seedlings to the VOCs of *Laccaria bicolor*, *Hebeloma cylindrosporum* and the beneficial endophyte *Serendipita indica*, induced root growth promotion under osmotic stress and regulated the expression of *WOX5.* This transcription factor preserves stem cell niche homeostasis and is also essential for maintaining the local auxin maximum in the root apex through the regulation of auxin transport and homeostasis (Savina et al., 2020). Although the regulation of *WOX* genes has not yet been studied in plants interacting with fungi, proliferation of lateral root primordia has been observed in mycorrhizal trifoliate orange seedlings under drought conditions (Liu et al., 2018; Zhang et al., 2022). The underlying mechanism involves the upregulation of auxin biosynthetic genes and transporters, suggesting that *WOX* family genes likely play a key role in communication between fungi and plants against abiotic stress.

Further evidence for the function of VOCs in the tolerance of plants to abiotic stress can only found for non-mycorrhizal fungi, for example, in plants under salt-stressed conditions with *S. indica* (Fraj and Werbrouck, 2023), *Fusarium oxysporum* and *Verticillium dahliae* (Li and Kang, 2018), *Trichoderma* spp (Jalali et al., 2017; Jiménez-Bremont et al., 2024). and *Penicillium aurantiogriseum* (García-Gómez et al., 2020).

In conclusion, despite the extensive information available on the role of mycorrhizal fungi in colonized roots under different abiotic stresses and the ongoing efforts to study VOCs from other microorganisms, the role of mycorrhizal VOCs in plant tolerance has been largely neglected. Elucidating the physiological and molecular roles of mycorrhizal VOCs in plant tolerance (**Figure 3**) will be crucial both to harness their potential as a sustainable biotechnological tool and to understand how these signals integrate into the overall plant response to different abiotic stresses.

## 5 Mycorrhizal VOCs in the tolerance of plants to biotic stress

Mycorrhizal VOCs are postulated to play a significant role in modulating plant responses to biotic stress by influencing defense signaling pathways and systemic resistance mechanisms against herbivores and pathogens (Razo-Belmán et al., 2023). Mycorrhizal VOCs is that they can exhibit direct antibacterial and antifungal properties (**Figure 3**), as previously shown in plants colonized by mycorrhizal fungi (Krywolap et al., 2011; Adeoyo et al., 2019; Bencherif et al., 2019).

Supporting this view, Osaki-Oka et al. (2019) demonstrated the role of VOCs from three ectomycorrhizal fungi (*Russula* aff. *anthracina*, *R. chloroides*, and *R. senecis*) in inhibiting the growth of various phytopathogenic fungi, identifying isovelleral as a major antifungal compound. Similarly, Abdulsalam et al. (2021) identified geosmin, limonene, and β-barbatene from the ECM fungus *Tricholoma vaccinum*, compounds previously described to possess antimicrobial activity (Bukvicki et al., 2013) (**Table 1**). Other volatile compounds with antifungal and broad-spectrum antimicrobial activity have been mainly described in non-mycorrhizal microorganisms, including hexane (Monsálvez et al., 2010; Salem et al., 2019), acetaldehyde (Avissar et al., 1990), anisole (Ojimelukwe and Adler, 1999; Yang and Liu, 2021), benzaldehyde (Ullah et al., 2015; Calvo et al., 2020), octanal (Hpoo et al., 2020; Li et al., 2021), and pentanal (Li et al., 2022; Zhang et al., 2024). These compounds have been shown in mycorrhizal volatile profiles from *Tuber* spp. and *Tricholoma* spp., among others (Cho et al., 2006; Splivallo et al., 2007; Guo et al., 2021; Li et al., 2021; Vita et al., 2015). These results pave the way for new lines of research into the role of mycorrhizal fungi’s VOCs in plant defense mechanisms.

Evidence on the role of mycorrhizal VOCs in the activation of plant defense mechanisms (**Figure 3**) as the Mycorrhiza-Induced Resistance (MIR) previously described in direct contact mycorrhizal-plant systems (Cameron et al., 2013; Delavaux et al., 2025) is completely lacking. However, VOCs from beneficial fungal symbionts have been proven to elicit Induced Systemic Resistance (ISR) against necrotrophic fungi such as *Botrytis cinerea*, effectively priming the plant immune system without physical contact (Contreras-Cornejo et al., 2014), what opens new lines of study of mycorrhizal fungi VOCs into plant defense mechanisms.

In summary, mycorrhizal VOCs are expected to coordinate with other key molecules in the multifaceted systemic defense strategy the mycorrhizal fungi-plant interaction. However, the information available is quite limited and mainly focused on the antibacterial and antifungal properties of mycorrhizal VOCs. The role of mycorrhizal VOCs in MIR resistance will need new approaches based on development of technology for the application of the molecules to be reproducible and viable for their application in field and green-house conditions.

## 6 Biotechnological applications of volatile compounds

Mycorrhizal fungi have been commonly applied in field and nursery conditions using different bioactive formulations of mixtures of fungi and microbial strains capable of establishing in the rhizosphere and forming direct interactions with plants (Bargaz et al., 2018; Koziol et al., 2024; Ghorui et al., 2025). Currently, the use of VOCs as a commercial product is still at the developmental stage (Razo-Belmán et al., 2023), since knowledge about the nature and mixture of VOCs to use, their concentrations, and the technology for their application requires further characterization.

**Figure 4:**
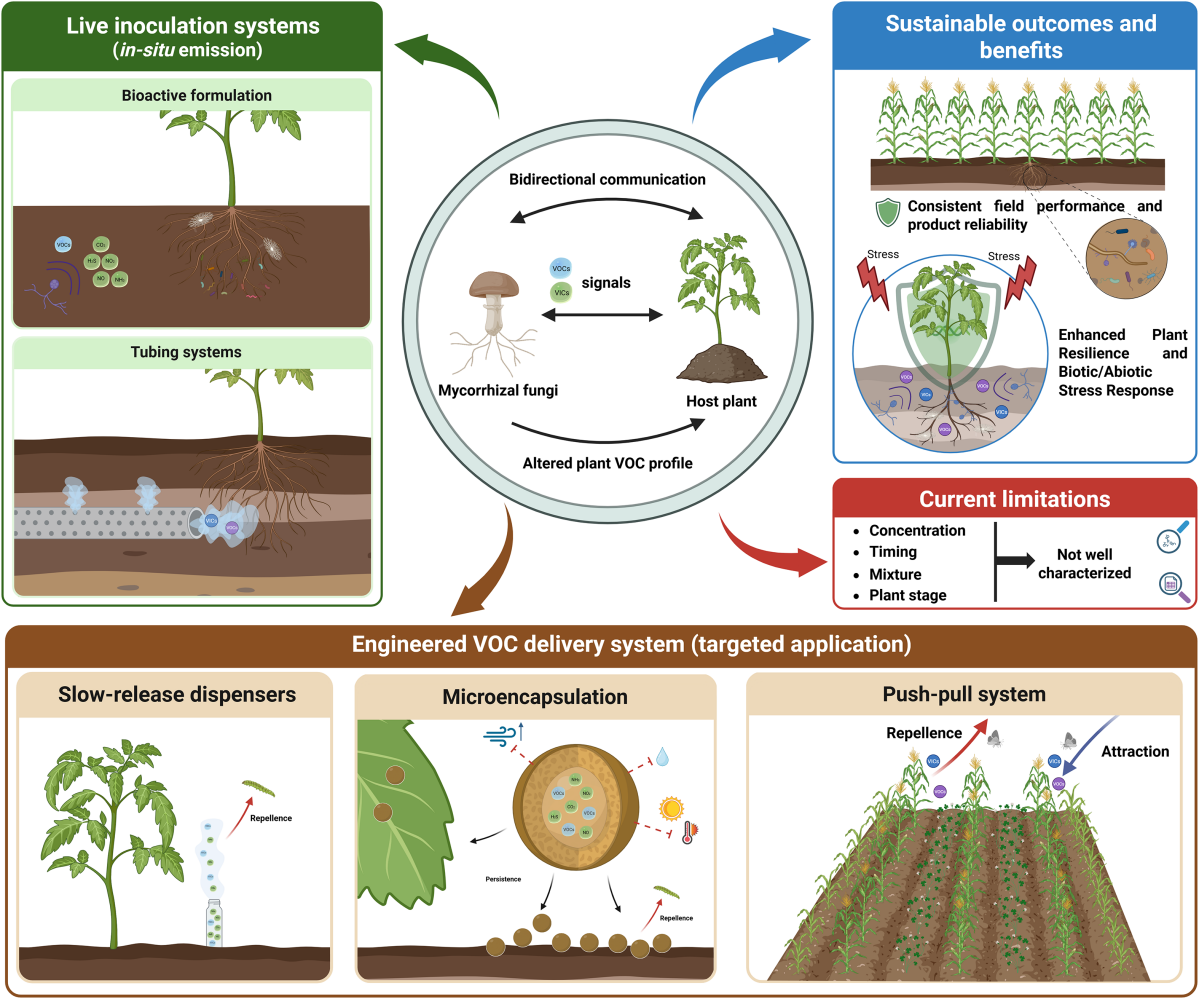
Biotechnological tools and applications of volatile compounds in sustainable agriculture.

Nevertheless, different biotechnological solutions have been proposed for the application of VOCs to plants (**Figure 4**). Moisan et al. (2021) used a tube-based system where soil-borne fungal inocula can be directly applied close to the plant roots, ensuring controlled and sustained emission of fungal VOCs within the rhizosphere to the plant. Most common is the application of isolated and purified VOCs to plants using dispensers (Veršić Bratinčević et al., 2023), microencapsulation (Alonso et al., 2021) or by the system `Push-Pull’ (Pickett et al., 2014; Zhang et al., 2023; Razo-Belmán and Ozuna, 2023). Dispensers allow for the slow and sustained release of VOCs, while microencapsulation creates a nanomaterial barrier to encapsulate target VOCs, preserving their properties and preventing loss due to chemical or physical degradation (Bakry et al., 2016). Finally, the “push-pull” system aims to both attract plants and herbivores that can repel certain insects away from crops (Pickett et al., 2014; Stenberg et al., 2015; Yi et al., 2019).

The different systems available for the application of VOCs are promising techniques that can serve as a base line for further technological development for the use and application of mycorrhizal VOCs under field and green-house conditions. The objective is to ensure a controlled emission of mycorrhizal VOCs while inducing the proper reaction of the plants to their related environment, with a consistent field performance that reinforces the role of mycorrhizal fungi beyond direct root and plant modulation.

## 7 Conclusions

Even though mycorrhizal fungi have been widely studied in direct contact with their hosts showing key roles in plant performance and in the tolerance of plants to biotic and abiotic stresses, the information on mycorrhizal VOCs, their composition and mode of actions and their role in the interplay of communications with plants is still scarce. The determination of the function and the main molecular insights that mycorrhizal VOCs are targeting to improve tolerance and defense in plants is a critical point to really understand mycorrhizal fungi-plant interactions and to advance in this field. This information, together with the diversity of mycorrhizal fungi and their capacity to produce a great arrangement of volatiles under different growing conditions, position them as a strong biotechnological tool for both agricultural and forestry crops, that not only will reduce dependence on agrochemicals but also fosters soil health and plant resilience, fully aligning with sustainable agriculture principles.
